# Effect of GenAI Dependency on University Students’ Academic Achievement: The Mediating Role of Self-Efficacy and Moderating Role of Perceived Teacher Caring

**DOI:** 10.3390/bs15101348

**Published:** 2025-10-02

**Authors:** Wenxiu Jia, Li Pan, Siobhan Neary

**Affiliations:** 1 Faculty of Education, Liaoning Normal University, Dalian 116029, China; jiawx111@163.com; 2 International Centre for Guidance Studies, University of Derby, Derby DE221GB, UK; s.neary@derby.ac.uk

**Keywords:** GenAI dependency, perceived teacher caring, media system dependency, self-efficacy, academic achievement

## Abstract

Generative artificial intelligence (GenAI) holds significant potential to enhance university students’ learning. However, over-reliance on it to complete academic tasks poses a risk to academic achievement by potentially encouraging cognitive outsourcing. Despite this growing concern and an expanding body of research on GenAI usage, the mechanisms through which GenAI dependency and perceived teacher caring affect their academic achievement and self-efficacy remain underexplored. Based on the theory of media system dependence, this study explores the mechanisms through which university students’ dependency on GenAI affects their academic outcomes, focusing on the mediating role of self-efficacy and moderating role of perceived teacher caring. A survey was conducted with 418 university students from Chinese public universities who had used GenAI for an extended period. The results revealed that GenAI dependency positively predicts false self-efficacy and negatively predicts academic achievement, exhibiting a significant Dunning–Kruger effect. Perceived teacher caring moderates the relationship between GenAI dependency and self-efficacy. High perceived teacher caring mitigates the Dunning–Kruger effect but has a weak moderating effect on academic achievement. These findings enhance the explanatory power of the media system dependency theory in educational contexts and reveal the pathways through which GenAI dependency and teacher caring affect learning processes and outcomes. This study expands the theoretical implications of teacher caring in the digital age and provides empirical evidence to aid higher education administrators in optimising AI governance and teachers in improving instructional interventions.

## 1. Introduction

The digital age has made knowledge acquisition easier and faster. However, the resulting information explosion has significantly increased our cognitive load, driving the need for more efficient information processing methods. Consequently, selectively outsourcing certain cognitive processes to external tools, such as generative artificial intelligence (GenAI), has become an important learning method and output approach for university students (Qu et al., 2024). GenAI has evolved from a mere assistant in knowledge production to a producer on par with humans, expanding learners’ cognitive channels and serving as an important supplement to formal education (Zhou & Peng, 2025). It demonstrates significant potential in enhancing learning efficiency, fostering inquiry-based learning, and providing personalized feedback (Cosentino et al., 2025). Although such reductive algorithms can achieve the preservation and arbitrary retrieval of knowledge, this knowledge does not undergo selective sampling through action and can severely disrupt learners’ mental processes. If students develop dependencies on GenAI, their cognitive abilities may atrophy or even short circuit, thereby affecting their academic development (L. Zhang & Xu, 2024). Therefore, research on the impact of GenAI dependency on university students across various dimensions, particularly the mechanisms related to academic development, holds significant value.

Academic achievement is a key indicator for evaluating students’ academic outcomes and the quality and equity of education across countries (OECD, 2016). In higher education, academic achievement refers to the benefits that students gain from education, including knowledge and cognitive improvements, such as academic performance (Serrano & Andreu, 2016), as well as improved comprehensive skills, such as teamwork, interpersonal communication, self-expression, and critical thinking (Liberal Education and America’s Promise, 2007). Academic achievement reflects students’ learning outcomes and developmental process, which are influenced by various factors, such as the educational environment and educators. Among these, the use of Generative AI (GenAI) presents a paradox. When utilized as an auxiliary tool, it can enhance learning efficiency and enrich the experience; however, when dependency shifts it toward a primary method, it risks hindering deep learning and skill development (Xu et al., 2025). For instance, students using GenAI for substitute thinking to avoid tackling difficult problems experience issues such as GenAI misuse and inadequate self-regulation (Zhai et al., 2024). This reduces their cognitive abilities, such as creativity and critical thinking skills, and severely affects the development of their emotional abilities, thereby decreasing the quality of higher education and placing students in a cognitive outsourcing trap (Vieriu & Petrea, 2025; Gerlich, 2025).

The media system dependency (MSD) theory posits that an individual’s degree of dependency on media is influenced by their goal structure, media resources, and social system (Ball-Rokeach & DeFleur, 1976). Within this dependency framework, individuals utilise the information and services provided by GenAI to achieve their goals, thereby enhancing their confidence in their problem-solving abilities (Anierobi et al., 2025). This enhanced confidence corresponds to the psychological construct of self-efficacy, which refers to an individual’s assessment of and belief regarding their ability to successfully perform specific behaviours to achieve their goals. Self-efficacy is a metacognitive evaluation of self-capability that influences students’ task selection, effort level, and persistence (Bandura, 1977). When students use GenAI to enhance their learning efficiency and solve problems in educational and research settings, their self-efficacy is promoted (J. Liang et al., 2023). However, overreliance on GenAI affects students’ cognition, behaviour, and attitudes, leading to the cognitive outsourcing and ability substitution phenomena (Tao et al., 2024). This weakens students’ autonomous learning, critical thinking, and analytical reasoning skills (Zhai et al., 2024), thereby affecting their academic achievement (Liu & Nesbit, 2024). 

According to the MSD theory, the audience–media–society triad forms an organic system, and teacher caring is an important social factor affecting university students’ GenAI dependency (Ball-Rokeach & DeFleur, 1976). As individuals increasingly rely on GenAI, its role and status in school education continue to increase. When teachers observe changes in students’ cognition, emotions, and behaviour, this triggers heightened attention to GenAI, including evaluating whether the knowledge it provides is universally applicable and authoritative and assessing the consequences of students’ GenAI dependency (Celik, 2023; Ning et al., 2024). Although teachers’ caring competence has been demonstrated to positively influence students’ academic achievement and self-efficacy (Riconscente, 2013), teacher caring is fundamentally a relational practice whose educational effectiveness depends on students’ perception and acceptance (Noddings, 2012). It is manifested through attentive listening, dialogic engagement, critical reflection, responsive feedback, and meaningful connection-building (Noddings, 2012). These behaviors must be recognized and internalized by students to be transformed into motivation for learning and development (Noddings, 2012). As emphasized by Muller (2001), only teacher caring that students can perceive is truly educational. Therefore, relying solely on teachers’ self-reported caring competence is insufficient to capture its actual educational impact. To more accurately reveal the mechanism by which teacher caring moderates the relationship between GenAI dependency and academic outcomes, this study shifts the focus of measurement from teachers’ self-assessment to students’ perceived teacher caring. This shift not only resonates with the core principle of perceptual agency within caring pedagogy but also captures from a systemic perspective how societal elements dynamically mediate the interdependence between audience and media through the intermediary node of students’ subjective perception. It thereby reveals the intricate interplay between technological and interpersonal factors within the educational ecosystem of the digital-intelligent era.

Although existing research has provided in-depth explorations of university students’ GenAI use, studies examining the relationship between GenAI and academic performance have primarily focused on its instrumental use (F. Martin & Borup, 2022; Nazaretsky et al., 2022). On one hand, most studies maintain a positive stance, emphasizing GenAI’s role in enhancing academic achievement and learning support. On the other hand, while some scholars acknowledge its value with underlying concerns, they have not systematically addressed the multidimensional negative impacts on students’ cognitive, emotional, and behavioral aspects when instrumental use shifts to substitute dependency. To address this gap, this study moves beyond the simplistic “tool-effect” paradigm by examining the mechanisms through which GenAI influences academic achievement as it shifts from an auxiliary tool to a dependency object. It introduces self-efficacy as a mediator and perceived teacher caring as a moderator to reveal the psychosocial pathways underlying technology dependency. Through this approach, this study aims to deepen critical understanding of the educational value of AI tools and expand the theoretical implications of teacher caring in the digital age, thereby providing a theoretical basis for AI education governance and teaching intervention practices. 

## 2. Literature Review and Hypothesis Development

### 2.1. GenAI Dependency and Academic Achievement

Based on the MSD theory, the relationship between GenAI dependency and students’ academic achievement can be analysed from the perspective of the media affecting the audience’s cognition, emotion, and behaviour. Higher media dependency is associated with a greater influence of the media on users’ cognition, behaviour, and attitudes. Moreover, the media can indirectly influence behavioural decisions by shaping social norm cognition (Ball-Rokeach & DeFleur, 1976).

University students’ GenAI use affects their cognition, manifesting in aspects such as knowledge utility, information acquisition, and problem solving. Previous studies have shown that during learning tasks, students are inclined to adopt solutions quickly generated by GenAI rather than invest in deeper cognitive efforts (Zhai et al., 2024; Xu et al., 2025; Yan et al., 2024). However, active cognitive effort during the learning process is a key element in improving academic performance (Lavrijsen et al., 2023). Therefore, although GenAI can promote educational development, vigilance regarding cognitive outsourcing is required (Kshirsagar et al., 2022).

University students’ deep engagement with GenAI affects their behavioral, manifesting in the technology’s progressive dominance over thinking patterns and behavioral approaches during learning and task execution. Overuse of GenAI can foster a preference for quick optimal solutions and cognitive shortcuts at the expense of deeper learning and creativity (Lin & Chen, 2024). This causes performance anxiety and reduces motivation for independent learning among students, as they may become less inclined to consult traditional resources or collaborate with peers (Lin & Chen, 2024), thereby hindering their academic achievement.

University students’ GenAI use involves their emotional needs, including feelings of efficacy, convenience, and trust (J. Liang et al., 2023; Biswas & Murray, 2024). Overreliance on GenAI may reduce face-to-face participation in the learning process, thereby posing the risks of social disengagement and relational poverty (Biswas & Murray, 2024). Although GenAI use can provide students with positive emotional experiences in problem solving, overreliance on it can lead to negative outcomes. Therefore, we propose the following hypothesis:

**H1.**  *GenAI dependency negatively predicts university students’ academic achievement.*

### 2.2. Mediating Role of Self-Efficacy 

The MSD theory suggests that the dependency relationship between individual audiences and the media is affected by two factors: goals and resources. Individuals rely on information resources controlled by the media to satisfy their needs and achieve their goals (Ball-Rokeach & DeFleur, 1976). Students use GenAI as a media-dependency tool, influencing their goal achievement and task completion by affecting the sources of self-efficacy, comprising mastery experiences, vicarious experiences, verbal persuasion, and physiological and emotional states (Wang & Chuang, 2024; L. Zhang & Xu, 2024). This effect on academic self-efficacy is complex and bidirectional, determining how it affects students’ academic achievement (Camacho-Morles et al., 2021).

When students successfully solve problems or complete tasks with the help of GenAI, this firsthand experience of success significantly enhances their self-efficacy in this domain. GenAI can help students quickly solve difficult problems, reduce homework stress, provide immediate feedback, reduce anxiety and frustration, and create a sense of ease and control (Alanazi et al., 2025), thereby maintaining or increasing their academic self-efficacy. This may enhance the positive effect of mastery goals and performance proximity to goals on academic achievement while reducing the negative effect of goal avoidance on academic achievement, thereby playing a supportive and protective role (Alhadabi & Karpinski, 2019). However, when students rely excessively on GenAI to directly generate answers or complete core tasks, this success cannot be internalised as a belief in their abilities. Students lack genuine mastery experiences, which may lead to false efficacy or an efficacy bubble. This erodes their foundational cognitive and skill-based knowledge and causes skill degradation and confusion regarding their beliefs in their abilities (Rodríguez-Ruiz et al., 2025). When students face scenarios requiring them to complete tasks independently, their false sense of efficacy ruptures, which negatively affects their motivation levels, learning effectiveness, learning outcomes, and autonomous learning performance (Guan et al., 2025).

GenAI dependency significantly affects university students’ self-efficacy levels in specific academic domains or tasks through the aforementioned complex mechanisms. This strongly impacts their subsequent academic behaviours and achievements. Therefore, understanding the nature and objectives of GenAI dependency, their effects on self-efficacy, and how these outcomes lead to differential academic achievement is essential. Therefore, we propose the following hypothesis:

**H2.**  *Students’ self-efficacy mediates the relationship between GenAI dependency and academic achievement.*

### 2.3. Moderating Effect of Perceived Teacher Caring

Based on the MSD theory, perceived teacher caring moderates the relationship between GenAI dependency and self-efficacy (Ball-Rokeach & DeFleur, 1976). This study focuses on students’ perceived teacher caring, which reflects how students perceive and feel about teachers’ caring behaviours. This study explores the effects of teacher caring on students’ cognitive, behavioural, and emotional dependency on external media, as well as their self-efficacy in this process.

When students perceive a high level of teacher caring, they feel that teachers maintain supportive attitudes and engage in caring practices during GenAI use (Jeon & Lee, 2023). In this process, teachers assess students’ cognitive and behavioural use of GenAI to determine whether they have developed dependency and employ critical thinking and reflective teaching to promote human–machine coordination, thereby addressing ethical issues, privacy protection, and GenAI misuse (Chan & Tsi, 2024). Studies have shown that teachers’ responsibilities in the digital age have expanded to include coordinating students’ reasonable and ethical use of GenAI and maintaining the humanistic aspects of education (Celik, 2023). This enriches the content and meaning of teacher caring and positively affects the development of students’ knowledge and abilities (Kim, 2023).

Conversely, if teachers fail to establish trust through effective caring and guidance during students’ GenAI use, negative emotional experiences (J. Luo, 2024), such as doubts about one’s capabilities, feelings of helplessness when faced with GenAI-generated content (Varol, 2025), and concerns about being perceived as dependent on GenAI, may occur. This can trigger negative emotions, such as anxiety and frustration, thereby weakening academic self-efficacy (Kundu & Bej, 2024).

Thus, students’ perceived teacher caring moderates their dependency on GenAI, as it can enhance or weaken the impact of GenAI dependency on their self-efficacy. Strong perceived teacher caring weakens the effect of GenAI dependency on students’ self-efficacy, whereas weak perceived teacher caring increases its effect. Therefore, we propose the following hypothesis:

**H3.**  *Perceived teacher caring moderates the relationship between students’ GenAI dependency and self-efficacy.*

The MSD theory suggests that people rely on the media to address ambiguous issues (Ball-Rokeach & DeFleur, 1976). In university students’ learning processes, self-efficacy serves as a crucial psychological resource for assessing their abilities to resolve ambiguous or complex problems and is a key factor influencing academic achievement (X. Huang et al., 2020). Although students’ GenAI use to address ambiguous problems provides some guidance, it negatively affects the development of their self-efficacy, thereby impacting the development of their overall capabilities (L. Zhang & Xu, 2024).

Teachers’ responses to students’ misuse of or overreliance on GenAI play a crucial role in shaping student development. Failing to make GenAI-dependent students feel cared for and guided and adopting harsh punitive methods, such as stern warnings, threats, or negative evaluations, may harm students’ emotional health, critical thinking, and social growth (Pillai et al., 2023). In GenAI-assisted learning environments, supportive and balanced teacher involvement is crucial to achieve positive student outcomes (Seo et al., 2024). Previous studies have shown that the integration of GenAI into education is transforming the role of teachers, who must become facilitators, guides, and moral managers of student learning in GenAI-enhanced learning environments (J. Zhang & Zhang, 2024; Moorhouse et al., 2024).

Therefore, this study focuses on students’ perceptions of teacher caring during GenAI use and its effect on their self-efficacy (Ball-Rokeach & DeFleur, 1976; Noddings, 2012). This enhances students’ cognitive abilities and helps them achieve human–machine coordination in GenAI use (Shekhar et al., 2018), thereby increasing their self-efficacy and academic achievement.

Based on the MSD theory, when students believe that teachers can provide them the resources required to achieve their goals, teachers become the objects of their new dependency relationships. The information resources held by teachers can influence students’ cognition, emotions, and behaviour. This can positively affect students’ knowledge and skill development as well as their ability to establish good relationships with others. In this context, perceived teacher caring enhances the role of self-efficacy, which may weaken the effect of students’ GenAI dependency on their academic achievement. Therefore, we propose the following hypothesis:

**H4.**  *Perceived teacher caring moderates the relationship between students’ GenAI dependency and academic achievement.*

**Figure 1:**
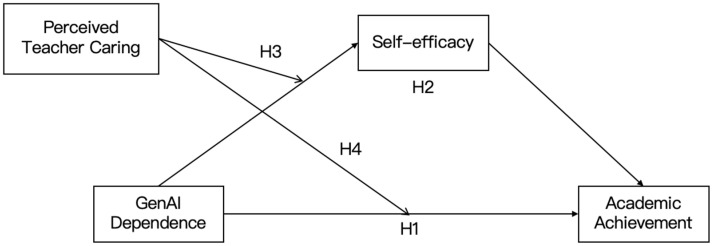
The proposed moderated mediation model.

The research model is presented in Figure 1.

## 3. Materials and Methods

### 3.1. Participants

The participants comprised full-time undergraduate students from multiple universities in Liaoning Province, China, who frequently utilized generative GenAI in their studies. Stratified random sampling was used to select participants across diverse genders, academic years, and disciplinary backgrounds to ensure sample representativeness. This study strictly adhered to the ethical guidelines of the Declaration of Helsinki. All participation was voluntary, with written informed consent obtained prior to data collection.

Questionnaires were administered through combined online and offline methods. The online survey was distributed via Wenjuanxing through an encrypted network link with IP addresses anonymized, while the offline survey was conducted in a controlled classroom setting using paper questionnaires, with researchers available to provide assistance. To enhance the scientific rigor of self-reported data, multiple procedural measures were implemented: all instruments featured random item sequences to mitigate order effects; scales for key constructs were presented in counterbalanced order to minimize common method bias; and explicit instructions emphasized response anonymity and the absence of right or wrong answers, encouraging honest self-assessments. Participants evaluated perceived teacher caring based on their experiences with instructional approaches, interpersonal interactions, and emotional support in daily academic life. Self-reported data were utilized to measure students’ self-efficacy and academic achievement.

Data cleaning was conducted to exclude invalid responses exhibiting straight-line patterns, internal inconsistencies, or implausibly short completion times. A total of 418 valid questionnaires were retained, yielding a valid response rate of 83.6%. Table 1 presents the distributions of gender, academic year, and disciplinary specialization among participants.

### 3.2. Measures

#### 3.2.1. GenAI Dependency

The Generative Artificial Intelligence Dependency Scale (GenAIDS) was developed through a multi-stage psychometric process grounded in Media System Dependency (MSD) theory. During the scale development process, we adhered to a standard pre-research protocol. Initially, based on the theoretical framework and relevant literature (S. Huang et al., 2024; S. Zhang et al., 2024; L. Zhang & Xu, 2024; Zhong et al., 2024), we generated an initial item pool comprising 20 items, covering three dimensions: affective, behavioural, and cognitive. Subsequently, a panel comprising two educational technology specialists and three psychology experts and doctoral candidates reviewed the content validity of the items. Based on their feedback, we revised the wording of certain items and removed four items exhibiting semantic overlap. Building upon this, we conducted interviews with 30 university students to ensure the clarity and comprehensibility of the item wording. Finally, a pilot study involving 150 university students enabled item analysis, leading to the removal of four items with inadequate discrimination (item-total correlation coefficients below 0.40). This resulted in a final 12-item scale covering the three dimensions.

The Affective Dependency dimension (4 items) measured students’ emotions and feelings when using GenAI, such as their sense of security, trust, satisfaction, and withdrawal symptoms (e.g., experiencing significant anxiety and uneasiness when unable to use GenAI and lacking confidence in one’s answers when unable to use GenAI). The Behavioural Dependency dimension (4 items) assessed students’ actions and habits when using GenAI (e.g., preferring to use GenAI even when capable of completing tasks independently and not considering alternative solutions when using GenAI). The Cognitive Dependency dimension (4 items) examined students’ thinking, judgment, knowledge acquisition, and evaluation when using GenAI (e.g., the efficiency of using GenAI in work and study and prioritizing GenAI when faced with complex problems requiring judgment or decision-making). Responses were rated on a five-point Likert scale (1 = strongly disagree; 5 = strongly agree), with higher scores indicating stronger GenAI dependency.

The confirmatory factor analysis results indicated a good fit (X^2^/df = 2.68, RMSEA = 0.055, GFI = 0.905, CFI = 0.924, TLI = 0.912, and IFI = 0.923). The standardised factor loadings for each item on its corresponding dimension ranged between 0.71 and 0.83 (see Appendix A), all exceeding the recommended threshold of 0.6, indicating that the scale possesses good convergent validity. Regarding validity, we further examined the scale’s convergent and discriminant validity. The composite reliability (CR) for the affective dependency dimension was 0.85, with an average variance extracted (AVE) of 0.59; the CR for behavioural dependency dimension was 0.84, AVE 0.57; and for cognitive dependency dimension, CR was 0.86, AVE 0.61. The CR values for all dimensions exceeded 0.70, and the AVE values surpassed the 0.50 benchmark, indicating the scale possesses sound convergent validity. Furthermore, the correlation coefficients between the three dimensions (0.52–0.61) were all below the corresponding AVEs (0.75–0.78), supporting the scale’s discriminant validity. Moreover, the overall internal consistency reliability of the scale was 0.91, with α coefficients for each dimension being 0.87 for affective dependency, 0.84 for behavioural dependency, and 0.82 for cognitive dependency, indicating excellent reliability. Taken together, these metrics demonstrate that the scale effectively reflects students’ degree of dependence on generative artificial intelligence.

#### 3.2.2. Self-Efficacy

This study employed the Chinese version (Y. Liang, 2000) of the Self-efficacy Scale, developed by Pintrich and De Groot (1990) (Pintrich & De Groot, 1990), to measure academic self-efficacy. The scale comprises 22 items in two dimensions: academic ability self-efficacy (11 items) and academic behaviour self-efficacy (11 items). Responses were rated on a five-point Likert scale (1 = strongly disagree; 5 = strongly agree), with higher scores indicating greater academic self-efficacy. In this study, the overall Cronbach’s alpha of the scale was 0.923.

#### 3.2.3. Perceived Teacher Caring

This study used the Teacher Caring Behaviour Questionnaire developed by Chinese scholar Hao Lei (H. Lei, 2014). We added and deleted specific items and adjusted the topics to focus on students’ perceptions of teachers’ caring behaviours (e.g., “when the students’ viewpoints conflict with mine, I will respect the students’ viewpoints.” revised as “when my viewpoint conflicts with the teacher’s viewpoint, the teacher will respect my viewpoint.”); however, we did not change the original dimensional structure. The scale comprises 14 items in three dimensions: responsibility (5 items), support (5 items), and inclusiveness (4 items). Responses were rated on a five-point Likert scale (1 = strongly disagree; 5 = strongly agree), with higher scores indicating higher levels of perceived teacher caring. In this study, the overall Cronbach’s alpha of the scale was 0.925.

#### 3.2.4. Academic Achievement

This study used educational outcome indicators from the Chinese National Survey on Student Engagement (NSSE) and grade point averages (GPA) to measure academic achievement. The NSSE-China scale was developed by Chinese scholars (Y. Luo et al., 2009) based on the NSSE scale (Pike, 2006) and tailored to the specific circumstances of Chinese university students. Educational outcomes primarily encompassed students’ self-assessment of knowledge and skills, critical thinking abilities, and self-awareness, comprising 11 items. Responses were rated on a five-point Likert scale (1 = strongly disagree; 5 = strongly agree), with higher scores indicating higher academic achievement. In this study, the overall Cronbach’s alpha of the scale was 0.77.

### 3.3. Data Analyses

Data were analysed using SPSS 26.0 and the macro program PROCESS v3.4.1. Descriptive statistics and correlation analyses were conducted using SPSS 26.0. PROCESS v3.4.1 was used to test the mediation and moderation models. Bootstrap sampling was repeated 5000 times with a confidence interval of 95%. Pearson correlation coefficient was used to examine the relationship between GenAI dependency and academic achievement. Building on this, we employed a moderated mediation model to assess the mediating role of self-efficacy in the relationship between GenAI dependency and academic achievement as well as the moderating role of perceived teacher caring. Demographic variables, comprising gender, grade, and major, were used as control variables.

## 4. Results

### 4.1. Common Method Bias

Based on procedural controls for potential common method biases (e.g., anonymous completion), the study adopted Harman’s single-factor test to examine common method bias in accordance with the recommendations of Podsakoff et al. (2003). Podsakoff and Organ (1986) argued that if the single-factor explained variance obtained through exploratory factor analysis does not exceed 50%, then the common method bias is not severe. D. Tang and Wen (2020) applied this test in China and revealed that the variance explained by a single factor should not exceed 40%. The current study showed that the variance explained by the first factor was 30.76%, which was below the 40% threshold. Therefore, this study did not exhibit significant common method bias.

### 4.2. Descriptive Statistics and Correlational Analysis

Table 2 presents the descriptive statistics and correlations for the key variables. GenAI dependency was relatively high (M = 3.26, SD = 0.79), while academic achievement (M = 2.11, SD = 0.65), self-efficacy (M = 2.22, SD = 0.61), and perceived teacher caring (M = 2.50, SD = 0.79) were all below the theoretical midpoint of 3.0. Correlation results showed that GenAI dependency was positively correlated with self-efficacy (r = 0.224, *p* < 0.01) and negatively correlated with academic achievement (r = −0.384, *p* < 0.01). Perceived teacher caring showed a significant negative correlation with GenAI dependency (r = −0.405, *p* < 0.01) and self-efficacy (r = −0.428, *p* < 0.01) but positively correlated with academic achievement (r = 0.159, *p* < 0.01). Although the negative correlation between perceived teacher caring and self-efficacy is counterintuitive, data verification confirmed its reliability. We posit that this does not imply that teacher caring diminishes self-efficacy; rather, it suggests that in contexts of high GenAI dependency, teacher caring may act as a “calibrating mechanism,” providing external feedback to counteract the self-perception bias induced by technology reliance, thereby aligning self-efficacy assessments more closely with actual competence. The subsequent moderation analysis and discussion will further elaborate on this mechanism. In addition, significant correlations were observed between gender, grade, major, and the key variables, so these demographic factors were controlled for in subsequent analyses.

### 4.3. Mediation Effect Test 

The study employed Model 4 in the SPSS macro developed by Hayes (2009). After standardising all the variables in the model, the study controlled for gender, grade, and major to examine the mediating effect of self-efficacy between academic achievement and GenAI dependency. The results are presented in Table 3. Grade positively predicted both academic achievement (β = 0.229, *p* < 0.05) and self-efficacy (β = 0.394, *p* < 0.05). Furthermore, GenAI dependency significantly predicted academic achievement (β = −0.281, t = −8.826, *p* < 0.001). Even after controlling for the mediating variable, GenAI dependency had a significant direct predictive effect on academic achievement (β = −0.239, t = −6.944, *p* < 0.001). Furthermore, GenAI dependency had a significant positive predictive effect on self-efficacy (β = 0.214, t = 9.003, *p* < 0.001), and self-efficacy had a significant negative predictive effect on academic achievement (β = −0.193, t = −2.966, *p* < 0.01). This indicated that self-efficacy played a significant mediating role in the process through which GenAI dependency affected academic achievement and that GenAI dependency significantly affected academic achievement. This suggested a partial mediation in the model. Path 1 represented the direct effect of GenAI dependency on academic achievement, whereas Path 2 represented the mediating effect of GenAI dependency → self-efficacy → academic achievement. Therefore, H1 and H2 were supported.

This study employed a bias-corrected nonparametric percentage bootstrap test, calculating 95% confidence intervals after 5000 resampling iterations to examine the total, direct, and mediating effects (Hayes & Scharkow, 2013). The results are presented in Table 4. The upper and lower bounds of the bootstrap 95% confidence intervals for the direct effect of GenAI dependency on academic achievement and the mediating effect of self-efficacy did not include 0; this suggested that GenAI dependency not only directly predicted academic achievement but also predicted academic achievement through the mediating effect of self-efficacy. The direct effect (−0.239) and mediating effect (−0.041) accounted for 85.26% and 14.74% of the total effect (−0.281), respectively.

### 4.4. Moderated Mediation Effect Test

We used Model 8 in the SPSS macro developed by Hayes (2009). The moderated mediation model was tested while controlling for gender, grade, and major. The results (Table 5 and Table 6) showed that after introducing perceived teacher caring as a moderator, the predictive effect of grade on academic achievement was further enhanced (β = 0.361, t = 9.947, *p* < 0.05), while its predictive coefficient for self-efficacy decreased (β = 0.375, t = 21.042, *p* < 0.05). In addition, the interaction term between GenAI dependency and perceived teacher caring significantly predicted self-efficacy (β = 0.052, t = 2.030, *p* < 0.05); but its moderating effect on academic achievement was not significant (β = −0.026, t = −0.704, *p* > 0.05). This indicates that perceived teacher caring moderated the first stage of the mediation path (GenAI dependency → self-efficacy) but did not significantly moderate the direct path from GenAI dependency to academic achievement. Therefore, Hypothesis 3 was supported, whereas Hypothesis 4 was not statistically supported.

**Figure 2:**
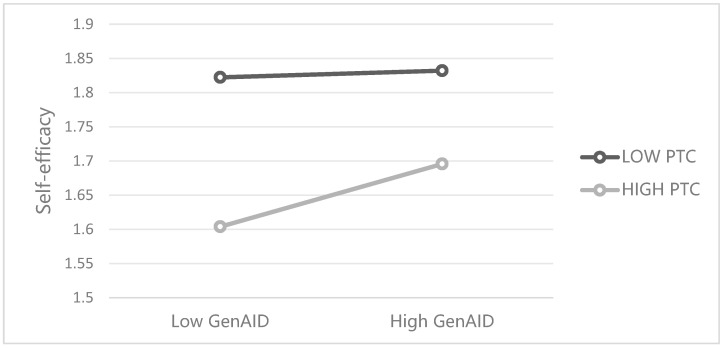
The mediating path coefficient. Notes: GenAID—Generative Artificial Intelligence Dependency; PTC—Perceived Teacher Caring.

To further validate the moderating effect of teacher caring, a simple slope analysis was conducted, dividing perceived teacher caring into two levels: low (M − 1SD) and high (M + 1SD). The results (Figure 2) showed that under high perceived teacher caring, the negative effect of GenAI dependency on self-efficacy was weaker, whereas under low perceived teacher caring, this negative effect was more pronounced. Moreover, as shown in Table 6, as the level of perceived teacher caring increased, the absolute value of the indirect effect of self-efficacy in the relationship between GenAI dependency and academic achievement also increased (from 0.013 to 0.025), suggesting that higher levels of teacher caring buffered the negative indirect effect of GenAI dependency on academic achievement through self-efficacy. In summary, the moderating role of teacher caring was primarily manifested in alleviating the negative impact of GenAI dependency on self-efficacy, thereby indirectly influencing academic achievement, rather than directly moderating the relationship between GenAI dependency and academic achievement.

## 5. Discussion

Based on the MSD theory, this study examined the mechanisms through which GenAI dependency affected university students’ self-efficacy and academic achievement, as well as the moderating role of perceived teacher caring in this process. The findings revealed that GenAI dependency positively impacted self-efficacy and negatively affected academic achievement. Additionally, the study found that perceived teacher caring moderated the relationship between GenAI dependency and self-efficacy among university students; however, its moderating effect on academic achievement was weak. This study contributes to and expands upon the existing research on GenAI dependency by offering insights for future studies in this area. 

### 5.1. Theoretical Implications

The results revealed that university students’ dependency on GenAI was significant and could negatively predict their academic outcomes. This study employed the MSD theory and found that when students’ use of GenAI reached the levels of cognitive, behavioural, and emotional dependency, the short-term, superficial objective achievements gained through GenAI’s shortcuts did not represent the internalisation of genuine knowledge and skills. Excessive GenAI dependency may impact students’ self-perception and self-concept formation (Yang et al., 2024), potentially leading to negative consequences such as cognitive outsourcing, skill substitution, social disengagement, and relational poverty. The phenomenon of media conditioning calls for a response from the social system to help students learn effectively and develop positively (J. Zhang & Zhang, 2024).

Furthermore, self-efficacy played a significant mediating role between GenAI dependency and academic achievement, although this mediation was qualified by the Dunning–Kruger effect. Specifically, students with lower self-control tend to use GenAI frequently to cope with academic demands or avoid task engagement, which can reinforce dependency (Rodríguez-Ruiz et al., 2025). However, this enhancement in self-efficacy does not translate into substantial improvements in academic achievement, indicating that students may experience cognitive biases in their self-assessment (Atir et al., 2024). According to the Dunning–Kruger framework, individuals lacking genuine competence often fail to accurately evaluate their own abilities, leading those with low ability to overestimate themselves (Kruger & Dunning, 1999). In this study, students who depend on GenAI to complete academic tasks generally lack an accurate assessment of their own abilities, leading to the development of a domain-specific “false self-efficacy” that masks their actual competence. This illusory confidence is not an overall increase in self-efficacy but is confined specifically to academic tasks where GenAI substitutes for students’ own intellectual effort, without reflecting genuine confidence gains in other life domains. Consequently, rather than fostering true capability, reliance on GenAI cultivates a misleading sense of competence within AI-assisted contexts, which ultimately undermines both academic performance and the development of authentic self-efficacy. This mechanism also serves as a caution for basic education, suggesting the need to foster metacognitive awareness and authentic self-efficacy in K–12 AI literacy education to prevent motivational distortions caused by technical dependency. These findings underscore the urgent need for pedagogically guided AI integration in higher education, specifically an approach that prioritizes metacognitive awareness and cognitive autonomy over passive technology reliance.

Moreover, the moderating role of perceived teacher caring on GenAI dependency and self-efficacy was significant. Research has shown that teachers’ caring capabilities can reduce the negative impact of GenAI dependency on students’ self-efficacy; however, their effect on academic achievement is limited. When students perceive high levels of teacher caring, teachers are more involved and provide more effective guidance during the students’ use of GenAI. This directly enhances students’ GenAI literacy and promotes positive changes in their self-assessment of their abilities, effectively mitigating the negative effects of GenAI dependency (Hagenauer et al., 2022). Additionally, this study found that perceived teacher caring acts as a calibrating mechanism for self-efficacy. By offering authentic feedback and support, teachers help students counter the false confidence resulting from GenAI dependency, leading to self-assessments that better reflect their true capabilities. This calibration effect not only serves as a crucial pathway to enhancing academic achievement but may also exhibit increasingly positive outcomes as students progress through higher grades and develop greater maturity. It also implies that integrating caring guidance into early AI literacy education can help younger students develop healthier and more autonomous attitudes toward technology use. This expands teachers’ role definitions in the digital age and advances theoretical discussions on the content of teacher caring (Sun, 2021; Song et al., 2022). This contribution reinforces the theoretical value of teacher caring in student learning and introduces a new moderator variable perspective for future research. It highlights the critical role of teachers’ caring education in students’ reasonable use of GenAI (Chan & Tsi, 2024). Additionally, this finding has significant implications for educational practice, emphasising that enhancing teachers’ caring is a key factor in successfully reducing students’ GenAI dependency.

### 5.2. Practical Contributions

This study revealed that students’ overreliance on GenAI had a significant negative impact on academic achievement, highlighting the potential negative effects of GenAI on the field of education. Therefore, educators and researchers urgently need to strengthen the monitoring and intervention of this phenomenon during the educational process. For instance, special attention should be paid to the construction of students’ belief in their true abilities, and vigilance should be exercised against the risk of false achievements masking a substantial decline in their sense of efficacy (L. Zhang & Xu, 2024). In this process, teachers should improve their understanding of GenAI applications and the new connotations of caring in the AI era and embed caring and guidance into interactions (Noddings, 2012). In every interaction with students, teachers should consciously convey support, trust, and expectations; create opportunities for students to experience genuine success; and guide students to conduct accurate self-assessments and effort attribution (Noddings, 2012). This will enhance students’ GenAI literacy and guide them to establish a healthy collaborative rather than exploitative relationship with GenAI and be vigilant against the cognitive outsourcing trap (Zhou & Peng, 2025). Moreover, educational administrators and policymakers should incorporate preventing overreliance and promoting responsible use into the top-level design of GenAI educational applications. For instance, teacher development and student assessment of curricula require the cultivation of AI ethics, critical use, and self-regulation skills (Karlen et al., 2023). Teachers should support the development of GenAI tools that enhance students’ metacognition and deep reflection and establish evaluation mechanisms to monitor cognitive engagement, genuine efficacy, and reflection depth during GenAI use (Yan et al., 2024).

Furthermore, self-efficacy served as a key bridge between GenAI dependency and academic achievement. This suggests that mere reliance on GenAI does not necessarily directly hinder academic achievement; its impact is primarily realised through its influence on students’ self-efficacy as an intermediary variable. Among these, the enhancement of genuine and stable self-efficacy is the core factor that promotes substantial progress in students’ academic achievement. Therefore, teachers should focus on enhancing students’ genuine self-efficacy, rather than merely relying on external tools. For instance, teachers can design instructional activities that guide students to succeed and set progressive challenge tasks, allowing them to experience growth in their abilities while using GenAI to assist in learning (Qi et al., 2025). Specifically, teachers can design "Generate–Reflect–Calibrate" cycle tasks to help students recalibrate self-efficacy perceptions distorted by GenAI use. First, ask students to use GenAI to generate a draft or solution. Then, guide them through structured reflection, such as summarizing the logical framework of the GenAI-generated content and identifying potential errors or limitations. Finally, have students revise and improve the output based on their own understanding. Throughout this process, teachers can provide metacognitive prompts and encouraging feedback to help students shift their sense of achievement from tool dependency to ability growth. Additionally, guiding students to conduct accurate self-assessments and attribution training is crucial, helping them recognise that effort and strategies, rather than simply relying on tools, are key to success (Mirriahi et al., 2025). Teachers should integrate GenAI tools into their traditional teaching methods. For instance, after using GenAI to generate learning materials, teachers can organise in-depth discussions and critical analyses or require students to engage in creative applications, ensuring that students are active constructors of knowledge rather than passive recipients (Xu et al., 2025). For policymakers, while promoting the application of GenAI in education, it is essential to prioritise the cultivation of students’ metacognitive abilities and genuine self-efficacy (Hwang et al., 2025). This includes supporting the development of GenAI tools that promote students’ reflective and self-monitoring abilities, as well as providing professional development training for teachers on strategies for cultivating self-efficacy.

Finally, the perceived level of teacher caring buffered the negative impact of GenAI dependency on self-efficacy and academic achievement, indicating that teacher caring is a key factor in mitigating GenAI learning risk. Therefore, educational institutions must prioritise enhancement as a core strategic objective. In the digital age, teacher caring requires educators to transcend the role of technical trainers and become “Educated Man” (J. R. Martin, 1981) who are deeply committed to the mission of nurturing students. Their educational teaching practices should be rooted in their educational philosophy and beliefs and focus on the creative transformation of their missions. As tacit knowledge that cannot be outsourced through technology, caring must be subtly conveyed through teacher–student interactions. Educators must practise the invisible caring teaching method (A. L. L. Tang & Walker-Gleaves, 2021). They should enhance their AI literacy to guide students in understanding the capabilities and ethical risks of GenAI, promote healthy human–machine coordination, and be vigilant against cognitive outsourcing. Additionally, they should embed caring and guidance into interactions, create authentic and successful experiences, guide students in accurate attribution, and provide cognitive adjustment and role model support (Newcomer, 2017). For example, in GenAI-assisted collaborative projects, teachers can incorporate group progress reflection sessions where students openly share their challenges and breakthroughs in using AI, followed by timely emotional affirmation and strategic feedback. Alternatively, through one-on-one learning planning meetings, teachers can help students set personal growth goals that utilize but also go beyond AI tools, making caring visible through sustained attention to students’ cognitive and psychological development. University administrators and policymakers need institutional support, such as time guarantees, recognition mechanisms, and a caring culture (Baig & Yadegaridehkordi, 2025). Professional development should be restructured to focus on caring abilities and reflect on the essence of education in the digital age. The evaluation system should be reformed to assess cognitive investment, reflective depth, genuine efficacy, and the quality of teacher–student interactions in the use of GenAI. Only by deeply embedding teacher caring—the human anchor—into digital education can we mitigate the risks of GenAI dependency and cultivate learners who possess technical adaptability, reflective ability, autonomy, and genuine academic competence.

### 5.3. Limitations and Future Directions

This study had several limitations. First, this study may be limited by its sample selection, as all participants were recruited from Chinese universities. The Chinese educational culture, which emphasizes respect for teachers and traditional hierarchies, may have shaped the moderating role of teacher caring observed in this study, characterized predominantly by guidance and responsibility. This cultural context could affect the generalizability of the findings to other educational settings. Therefore, future research should include participants from diverse cultural backgrounds and extend to different educational stages to examine the cultural specificity and stability of teacher caring’s moderating effect between GenAI dependency and academic achievement, thereby enhancing the cross-cultural applicability of the research model.

Second, this study employed a cross-sectional data analysis, making it difficult to precisely capture causal relationships, particularly the long-term effects of GenAI dependency on student achievement. Although the study made efforts to minimise errors in data collection, it inevitably had limitations, such as measuring the long-term effects of teacher caring. Therefore, future research should adopt a longitudinal study design to track students’ long-term performance in a GenAI-dependent learning environment and continuously explore the mechanisms through which GenAI dependency influences university students.

Finally, this study relied primarily on questionnaire surveys, with all core variables measured through self-reporting. Although this method offers advantages in terms of efficiency and ease of administration in large-scale data collection and aligns with the study’s focus on students’ subjective experiences, its limitations cannot be overlooked. On one hand, relying on a single source of quantitative data makes it difficult to completely avoid common method biases such as social desirability and response errors. On the other hand, self-reported data may not fully or objectively reflect actual academic performance. To enhance the reliability and ecological validity of the findings, future research could introduce external data such as official institutional grades and teacher evaluations for triangulation, combined with qualitative methods like interviews and classroom observations. This would allow for a more thorough and multidimensional understanding of the underlying mechanisms and real-world contexts through which GenAI dependency influences student development. Moreover, to advance the standardization and application of the measurement tool, future studies could build upon the validity established in this research to provide more direct evidence for discriminant validity through tests of competing models in new samples.

## 6. Conclusions

This study investigated the effect of GenAI dependency on students’ academic achievement and revealed the mediating role of self-efficacy and moderating role of perceived teacher caring. The results indicated that GenAI dependency negatively affected students’ academic achievement and indirectly influenced it by increasing false self-efficacy. Perceived teacher caring played a crucial moderating role in this process. Based on these findings, educators should continuously expand the scope of teacher caring and focus on technical aspects. This will help students transition from an exploitative to a collaborative GenAI usage pattern, fostering authentic ability beliefs and reflective thinking. Future research could further explore the deeper mechanisms of GenAI dependency in student learning processes, as well as other potential influence mechanisms, thereby providing comprehensive theoretical support and practical guidance for GenAI-driven education and student human–machine coordination in the digital age. 

## Figures and Tables

**Table 1:** Sample basic information (N = 418).

		Frequency	Percent (%)
Gender	Male	175	41.9
	Female	243	58.1
Grade	Freshman	118	28.2
	Sophomore	102	24.4
	Junior	97	23.2
	Senior	101	24.2
Major	Arts	159	38.0
	Science	117	28.0
	Engineering	142	34.0

**Table 2:** Means, standard deviations, correlations, and reliability of study variables.

	Mean	SD	1	2	3	4	5	6	7
1. Gender	1.58	0.494	1						
2. Grade	2.43	1.139	0.504	1					
3. Major	1.96	0.849	−0.599	−0.337	1				
4. GenAID	3.26	0.79	−0.057	−0.120 *	−0.113 *	1			
5. AA	2.11	0.65	0.359 **	0.779 **	−0.192 **	−0.384 **	1		
6. SE	2.22	0.61	0.415 **	0.779 **	−0.333 **	0.224 **	0.222 **	1	
7. PTC	2.50	0.79	0.408 **	0.838 **	−0.295 **	−0.405 **	0.159 **	−0.428 **	1

Notes: GenAID—Generative Artificial Intelligence Dependency; AA—Academic Achievement; SE—Self-efficacy; PTC—Perceived Teacher Caring; * *p* < 0.05; ** *p* < 0.01.

**Table 3:** Regression Analysis of Variable Relationships in the Mediation Model.

Variable	Academic Achievement			Academic Achievement			Self-Efficacy		
	β	t	95%CI	β	t	95%CI	β	t	95%CI
Gender	−0.073	−1.086	[−0.206, 0.059]	−0.071	−1.045	[−0.205, 0.063]	−0.011	−0.211	[−0.111, 0.089]
Grade	0.375	10.500 *	[0.305, 0.445]	0.299	11.930 *	[0.250, 0.348]	0.394	21.037 *	[0.357, 0.431]
Major	−0.016	−0.423	[−0.088, 0.057]	−0.007	−0.178	[−0.079, 0.066]	−0.046	−1.676	[−0.100, 0.008]
GenAID	−0.239	−6.944 ***	[−0.307, −0.172]	−0.281	−8.826 ***	[−0.343, −0.218]	0.214	9.003 ***	[0.167, 0.261]
SE	−0.193	−2.966 **	[−0.322, −0.0655]						
R^2^	0.413			0.401			0.621		
F	57.991 **			68.988 **			169.494 **		

Notes: GenAID—Generative Artificial Intelligence Dependency; SE—Self-efficacy; * *p* < 0.05, ** *p* < 0.01, *** *p* < 0.001.

**Table 4:** The Mediating Effects of GenAI Dependency, Self-efficacy, and Academic Achievement.

	Effect	BootSE	BootLLCI	BootULCI	Relative Effect Size
Total effect	−0.281	0.032	−0.343	−0.218	
Direct effect	−0.239	0.035	−0.307	−0.172	85.26%
Indirect effect	−0.041	0.015	−0.074	−0.013	14.74%

Notes: Boot standard error, lower limit of Boot CI, and upper limit of Boot CI refer to the standard error, lower limit, and upper limit of the 95% confidence interval of the indirect effect estimated by the bootstrap method with deviation correction, respectively. All values are rounded to three decimal places, and the same applies below.

**Table 5:** Test of the Moderated Mediation Model.

Variable	Self-Efficacy			Academic Achievement		
	β	t	95%CI	β	t	95%CI
Constant	1.807	6.895 ***	[1.292, 2.323]	2.030	5.180 ***	[1.259, 2.800]
Gender	0.001	0.014	[−0.093, 0.094]	−0.078	−1.153	[−0.210, 0.055]
Grade	0.375	21.042 *	[0.340, 0.410]	0.361	9.947 *	[0.290, 0.433]
Major	−0.033	−1.296	[−0.084, 0.017]	−0.018	−0.495	[−0.090, 0.054]
GenAID	0.006	0.090	[−0.130, 0.142]	−0.156	−1.594	[−0.348, 0.036]
SE				−0.141	−2.019 **	[−0.278, −0.004]
PTC	−0.347	−4.183	[−0.510, −0.184]	0.152	1.269	[−0.084, 0.387]
GenAID × PTC	0.052	2.030 *	[0.002, 0.102]	−0.026	−0.704	[−0.097, 0.046]
R^2^	0.6714			0.419		
F	139.96 ***			42.267 ***		

Notes: GenAID—Generative Artificial Intelligence Dependency; SE—Self-efficacy; PTC—Perceived Teacher Caring; * *p* < 0.05, ** *p* < 0.01, *** *p* < 0.001.

**Table 6:** Direct Effects and Mediating Effects of Different PTC.

	PTC	Effect	BootSE	BootLLCI	BootULCI
Direct Effect	Low (M − 1SD)	−0.200	0.046	−0.290	−0.110
	Medium (M)	−0.220	0.036	−0.290	−0.150
	High (M + 1SD)	−0.240	0.046	−0.330	−0.150
Indirect Effect	Low (M − 1SD)	−0.013	0.010	−0.036	0.002
	Medium (M)	−0.019	0.012	−0.045	0.002
	High (M + 1SD)	−0.025	0.015	−0.059	0.003

## Data Availability

The data presented in this study are available on request from the corresponding author due to privacy restrictions.

## Appendix A

**Table A1:** GenAIDS Confirmatory Factor Analysis Standardized Factor Loading Table.

Dimension	Item	Standardized Factor Loading
Affective Dependency	I feel noticeably anxious or unsettled when I am unable to use Generative AI.	0.75
	Without the assistance of Generative AI, I lack confidence in the answers I arrive at through my own thinking.	0.78
	I trust that Generative AI can reliably protect my personal privacy and data security.	0.71
	I find the results and explanations provided by Generative AI to be trustworthy.	0.82
Behavioral Dependency	Even for tasks I am capable of completing independently, I give priority to using Generative AI to solve them.	0.81
	After obtaining an answer from Generative AI, I usually do not actively seek alternative solutions.	0.76
	In my studies or work, using Generative AI has become a natural and primary choice.	0.72
	I have noticed that the amount of time I spend on Generative AI is increasing.	0.74
Cognitive Dependency	When dealing with complex problems, my first thought is to seek ideas or answers from Generative AI.	0.83
	I firmly believe that using Generative AI significantly enhances my learning and work efficiency.	0.79
	Since information can be quickly obtained through Generative AI, I believe it is no longer necessary to expend effort memorizing large amounts of knowledge.	0.74
	I worry that my ability to solve problems independently would be insufficient without Generative AI.	0.77
